# Clinical application of ultrasound-guided percutaneous microwave ablation for benign breast lesions: a prospective study

**DOI:** 10.1186/s12885-019-5523-6

**Published:** 2019-04-11

**Authors:** Wei Zhang, Zhan-Qiang Jin, Masoud Baikpour, Jian-Min Li, Hui Zhang, Ting Liang, Xiao-Ming Pan, Wen He

**Affiliations:** 1 Department of Ultrasound, Beijing Tian Tan Hospital, Capital Medical University, No. 119, West Road of South 4th Ring Road, Fengtai District, Beijing, 100160 China; 2 Department of Ultrasound, Affiliated Hospital of Guilin Medical University, Guilin, China; 3 Department of Ultrasound, The 3rd Affiliated Hospital of Guangxi Medical University, Nanning, China; 4 School of Medicine, Tehran University of Medical Sciences, Tehran, Iran; 5 Department of Thyroid and Breast Surgery, The 3rd Affiliated Hospital of Guangxi Medical University, Nanning, China

**Keywords:** Benign breast lesion, Microwave ablation, Efficiency, Safety

## Abstract

**Background:**

Background: Benign breast lesions are the most common diseases in adult women, which have been treated with minimally invasive therapies in recent years. Little is known about the feasibility of Microwave ablation (MWA) for benign breast lesion treatment. The primary aim of this prospective study was to evaluate the safety and efficiency of MWA as a potential therapeutic option for benign breast lesions in a single-center cohort study.

**Methods:**

Women with possibly benign breast lesions based on an ultrasound (US) assessment who were scheduled to undergo MWA between November 2014 to July 2018 were included in the study. The patients underwent conventional US to measure the size of the lesion, Doppler US to assess the vascularity of the lesion, elastography to evaluate the stiffness of the mass, core needle biopsy of suspicious lesions, contrast-enhanced US to help determine the treatment plan and eventually MWA of the lesion. Lesions were followed at one, three, six, twelve and eighteen months after treatment to with the same imaging modalities.

**Results:**

A total of 314 women aged 17 to 69 years old (mea*n* = 36.9 ± 9.9 years) with 725 benign breast lesions (mean of maximum diameter = 10.86 ± 5.40 mm) were included. The frequency of palpable mass, pain and nipple discharge significantly decreased after treatment. Complete ablation rate was 97.8%, immediately after ablation, which increased to 100% after supplementary ablation of the 15 cases with incomplete ablation. Blood flow classification and lesion’s volume also showed a significant decrease, while both volume reduction ratio and disappearance rate significantly increased following treatment. The elasticity score of the lesions showed fluctuations across different follow-up intervals. None of the patients experienced major complications and the 1% who had mild symptoms were successfully treated.

**Conclusion:**

MWA treatment is shown to be safe and efficient and has the potential to be considered as an alternative first line treatment for benign breast lesions.

## Background

Benign breast lesions such as fibroadenoma and mammary adenosis are amongst the most common diseases in adult women, usually presenting as multiple and bilateral masses [1, 2]. Although surgical intervention is considered as the common treatment, it can be associated with complications such as hemorrhage, hematoma and poor cosmetic outcomes including formation of scar, asymmetry of the breasts, etc. [3]. In this regard, major attention has been drawn to application of minimally invasive methods as potential alternatives for the treatment of benign breast lesions, such as percutaneous ablation and the mammotome system, which has been approved for removal of small fibroadenomas [4].

Ultrasound (US) -guided percutaneous ablation is a non-surgical minimally-invasive method that can be performed in both outpatient settings and ambulatory operating rooms, with high success rates and low complication rates. There are several ablation techniques such as radiofrequency, microwave, laser and high-intensity focused US, of which radiofrequency ablation (RFA) is considered to be the most promising technique for treatment of small breast malignancies and benign breast lesions [5–9].

Microwave ablation (MWA) has been shown to have a higher efficiency in heating the target tissue compared to RFA, which is due to the difference in heating mechanisms of the two techniques; microwave heating is a result of dielectric heating, while radiofrequency is resulting from Joule heating, in which the tissue is heated through friction and thermal conduction from the electrode and it is strongly influenced by the tissue’s impedance. In comparison between the two techniques, MWA has been shown to not only provide a larger ablative volume, but also a smoother coagulation margin [10]. MWA has already shown promising results in treatment of kidney and liver lesions, but few studies have evaluated its value in the treatment of benign breast lesions. Accordingly, the present study investigated the outcomes of US-guided MWA treatment in patients with benign breast lesions to assess the feasibility and efficiency of this method.

## Methods

### Study design and sample population

The University Ethics Committee approved this prospective study and a written informed consent was obtained from all the included patients. The target population for the study included women who had an US assessment of a breast lesion between November 2014 to July 2018, following pain or palpation of a mass in the breast. The patients were diagnosed to have a possibly benign breast lesion of categories 2 and 3 per the breast US lexicon of breast imaging reporting and data system (BI-RADS) [11].

Inclusion criteria for the study were determined as: (1) diagnosis of a benign breast lesion according to US assessment; (2) low probability of malignancy (BIRADS 2 and 3); (3) ineligibility for surgical resection as a result of the patient’s physical condition, cosmetic considerations or refusal of the patient for surgery. Exclusion criteria included: (1) history of a serious coagulation disorder; (2) acute or active infectious disease; (3) severe hypertension, diabetes and cardiopulmonary dysfunction; (4) large lesion (diameter > 3 cm); (5) pregnancy and lactation.

### Ultrasound techniques and measurements

All the US assessments were performed by the HI VISION Ascendus (Hitachi Medical, Tokyo, Japan) ultrasound equipment with a linear probe (L74 M).

- *Conventional US*

The maximum diameter of the lesion in 3 axes was measured on the B-mode US images and their volumes were estimated by the following formula, assuming an ellipsoid shape for the lesions:

$$ Volume=\frac{length}{2}\times \frac{width}{2}\times \frac{heigth}{2}\times \frac{4\pi }{3} $$

The volume-reduction ratio (VRR) was calculated by the following equation:

VRR = (initial volume – final volume) × 100/initial volume

- *Doppler US*

Based on the lesions’ Doppler US assessments the intralesional blood flow for each lesion was rated on a scale of 0) no flow, 1) minimal, 2) moderate and 3) marked, according to the criteria proposed by Adler et al. [12].

- *Elastography*

Strain elastography images were generated by freehand manual compression. The region of interest (ROI) box extended from the subcutaneous fat layer to the superficial portion of the pectoralis muscle layer. Sonographers obtained optimal elastography images of the lesions, optimal being defined as either presence of color homogeneity within the ROI or stable waveform curve in the pressure indicator displayed on the screen. The color code on elastography images ranged from red, green to blue, showing areas of greatest strain (i.e., softest component), moderate strain and no strain (i.e., hardest component). Elasticity of the lesions was scored on a scale of 1 to 5 according to the scoring system established by Itoh et al. [13].

- *US-guided core needle biopsy (CNB)*

Following the above-mentioned assessments of the lesions, US-guided CNB was performed, the samples were reviewed by pathologists and the histopathological diagnosis of the lesions was established.

- *Contrast-Enhanced US*

Contrast-enhanced US (CEUS)allows immediate assessment of ablation outcome, and intraprocedural CEUS can effectively decrease the need for additional ablative sessions [14] After recording the histopathological diagnosis and before performing the ablation, CEUS was performed on all patients. Standard machine settings were used, with mechanical index set at 0.05 to 0.08, and the focus placed deeper than the lesion plane to prevent destroying the microbubbles. SonoVue (BR1, Bracco Imaging, Milan, Italy) was used as the contrast agent, which was supplied as a sterile, lyophilized powder contained in a septum-sealed vial. A suspension of the contrast agent was prepared by adding 25 mg of the powder to 5 ml normal saline. The contrast bolus was then injected manually at a flow of approximately 1 ml/s, followed by a 5-ml saline solution flush, via a 20-gauge intravenous cannula placed in an antecubital vein by a trained nurse. The imaging data acquired by CEUS were continuously stored in the ultrasound machine over a period of 120 s, using a time-triggered acquisition technique. The enhanced features of peripheral and internal parts of lesion were observed.

### Contrast-enhanced magnetic resonance imaging (CE-MRI)

Patients with lesions with a maximum diameter greater than 2 cm, multiple lesions in one breast, or lesions with irregular shapes, along with subjects whose CEUS images had low resolutions underwent CE-MRI. In our study, The imaging of MRI was performed with the AGNETOM Verio 3.0 T (Siemens, Erlangen, Germany), and Gadopentetate dimeglumine (Consun, Guangzhou, China) was used as a contrast agent.

### Microwave system, ablation procedure and follow up

US-guided percutaneous MWA was then performed under the guidance of the same US machine. The microwave system (KY-2000,Canyon Medical, China) consisted of a microwave generator (2450 MHz), a hollow water-cooled-shaft antenna (with a 3-mm active tip of microwave transmission).

The output power of the system was set at 20 to 50 W. In order to achieve the best treatment outcome, the optimal ablation scheme was designed based on the number of lesions, their locations, blood flow, size, shape and the findings of the preoperative CEUS, following these instructions: (1) If the patient has multiple nodules in the same breast that need to be treated, the number of skin puncture points should be minimized by trying to use one puncture point to ablate multiple lesions. (2)If possible, the puncture point should be set at the periphery of the mammary gland rather than the areola area, and preferably 1.5–2 cm away from the tumor. (3) If the tumor has large supporting vessels, it is recommended to coagulate the vessels first, which can improve the thermal efficiency [15]. (4) The moving shot technique should be utilized for large and irregular lesions [16].

**Fig. 1:**
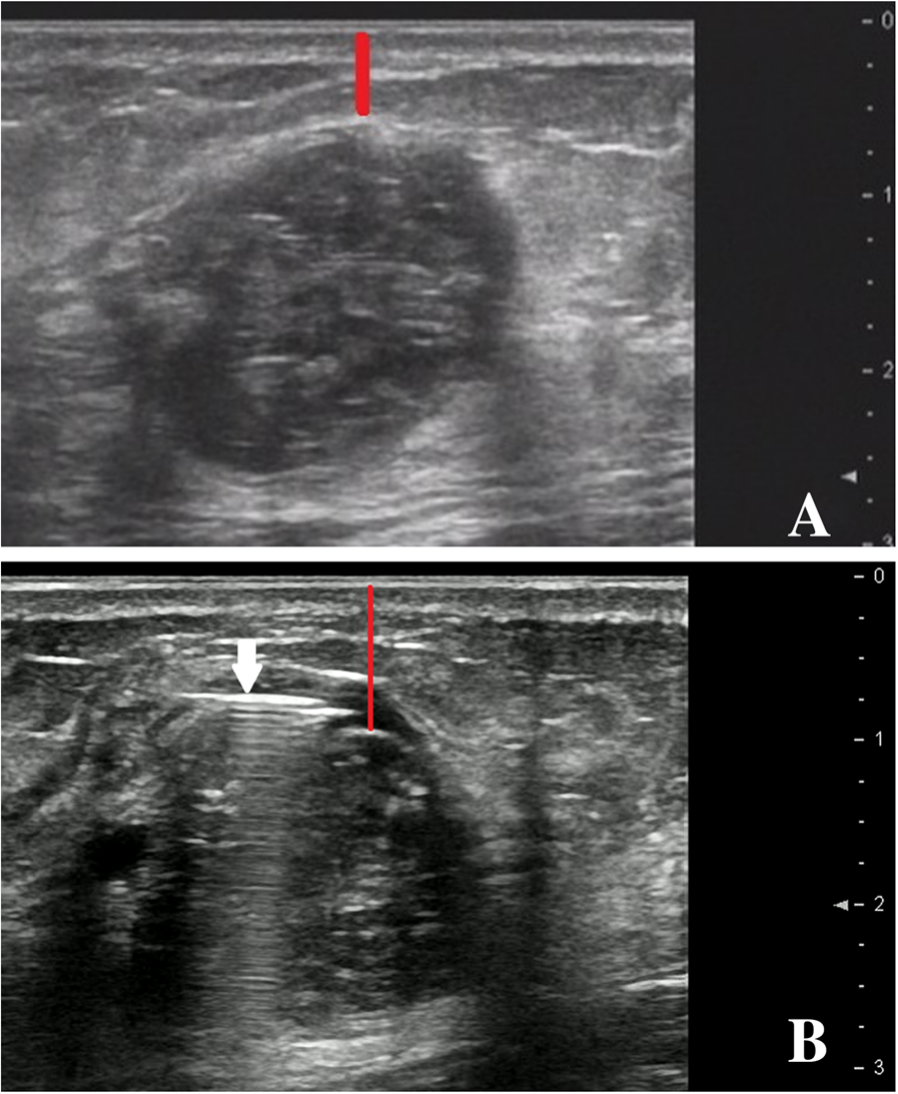
A 28-year-old woman with fibroadenoma; (**a**) the distance between the lesion and the skin is less than 5 mm, (**b**) the spacer fluid was injected into the subcutaneous space increasing the distance between the lesion and the skin; the arrow is pointing to the needle

**Fig. 2:**
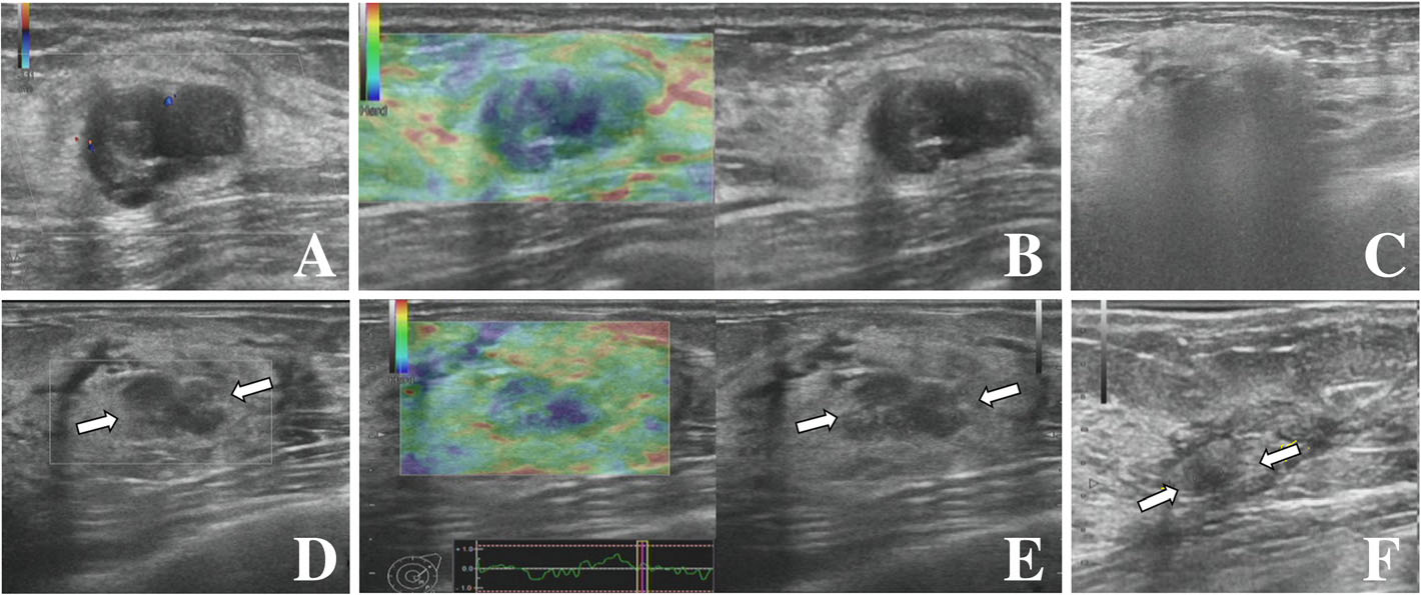
A 31-year-old woman with a BI-RADS 3 fibroadenoma. (**a**) Conventional US detected a hypoechoic, solid mass with a well-defined boundary and a transverse axis diameter of 16 mm located in the left breast; color Doppler imaging showed the lesion to be vascularized (Grade II); (**b**) on elastography, the lesion was classified as Itoh score 3; (**c**) gray-scale imaging during ablation showed a hyperechoic mass with ill-defined boundaries; (**d**) conventional US performed at the 3-month follow up showed a heterogeneous hypoechoic mass with a transverse axis diameter of 10 mm; (**e**) on elastography at the 3-month follow up, the lesion was found to be softer than before ablation with a Itoh score of 2; (**f**) the lesion is hardly visible on conventional US imaging at the 12-month follow up

All the procedures were performed by interventional radiologists with more than 4 years of experience in breast ultrasound, CNB and microwave ablation. The patient was positioned in the supine or latericumbent position, the area was sterilized, and local anesthesia was injected. In cases where the distance between the lesion and the skin and/or pectoral fascia was less than 5 mm, a solution (5% saline) was injected into the subcutaneous and/or retromammary space to widen the distance between them in order to avoid injury to the skin and/or fascia (Fig. 1). Since it is difficult for the blunt tip of the antenna to penetrate the skin, a 2 mm incision was made, and the water-cooled-shaft of the antenna was introduced percutaneously under US guidance. The ablation was started under real-time US monitoring. The microwave irradiation lasted between 3 to 8 min, depending on the size and blood supply of the lesion. During the ablation procedure, a nurse monitored the microwave machine and looked for any possible complications that could develop in patients. Half an hour after the MWA, CEUS was performed again to assess the efficiency of the treatment. Cases with enhancement in the interior and periphery of the lesions underwent supplementary ablation. Complete ablation was also assessed according to the results of the post-ablation CEUS and/or contrast enhanced MRI and/or pathology at 24 h post-ablation. Figure 2 depicts the different steps for one case.

Lesions were followed at one, three, six, twelve and eighteen months after the MWA to further evaluate the therapeutic effects using the gray-scale and Doppler US, and strain elastography. The outcome measures at each interval included blood flow classification, estimated volume of the lesion and strain elasticity score. VRR was also calculated and recorded for each follow up. The patients were also asked about presence of palpable mass, pain and nipple discharge at each follow up. Development of post-intervention complications such as pricking, skin scalding, local erythema and swelling, and fat liquefaction was also evaluated and recorded for each lesion.

### Variables

Evaluated variables in this study included the pathology and position of the lesions, their distance from skin and pectoralis muscle, the power, time, energy and unit energy of the ablation, and the complications associated with MWA. Blood flow classification according to Color Doppler Flow Imaging, CEUS and/or MRI, volume of the lesion, volume reduction ratio, disappearance rate and elasticity scores were also assessed for all the lesions before the ablation and at the five determined follow up intervals.

### Statistical analysis

SPSS 22.0 for Windows® software package (SPSS Inc., Chicago, IL, USA) was used for statistical analysis [17]. Descriptive statistics were calculated for the entire patient cohort. Qualitative variables are summarized as frequency (percent) and quantitative variables are summarized as median (25th percentile – 75th percentile). Chi-squared test and Kruskal Wallis test were used to assess the changes in the qualitative and quantitative variables after treatment, respectively. A *p* value of less than 0.05 was considered to be statistically significant.

## Results

### Descriptive statistics of patients

A total of 314 women aged 17 to 69 years old (mean = 36.9 ± 9.9 years) with 725 benign breast lesions (mean of longest diameter = 10.86 ± 5.40 mm) were included in the present study. The number of lesions in one patient ranged from 1 to 17 with a median of 2 lesions per patient. All patients provided information about the symptoms of palpable mass, pain and nipple discharge before the treatment. Frequencies of these symptoms were also investigated after treatment and the results were reported based on the latest follow up of the patients. Accordingly, the frequency of palpable mass decreased from 29.8 to 11.4% (216/725 to 76/668; *p* < 0.001, frequency of patients who felt breast pain decreased from 18.8 to 7.4% (59/ 314to21 /284; *p* < 0.001) and the percentage of patients with nipple discharge decreased from 13.4 to 3.9% (42/314 to 11/284; *p* < 0.001).

### Descriptive statistics of lesions

Descriptive statistics of the 725 lesions included in the analyses are presented in Table 1. Accordingly, the majority of the lesions were diagnosed as adenosis (55.4%) and fibroadenomas (40.3%). Most of the lesions were located in the upper outer quadrant of the breast (40.6%) and the median distance of the lesion to the skin and pectoralis muscle were 7.0 (4.0–10.0) and 4.0 (1.5–8.0), respectively.

**Table 1:** Descriptive statistics of the lesions included in the study

Variable	Descriptive statistics^a^
Pathology^b^	
Adenosis	220 (55.4%)
Fibroadenoma	160 (40.3%)
Intraductal papilloma	9 (2.3%)
Other	8 (2.0%)
Position	
Upper Outer Quadrant	294 (40.6%)
Upper Inner Quadrant	206 (28.4%)
Lower Inner Quadrant	92 (12.7%)
Lower Outer Quadrant	133 (18.3%)
Distance to skin	7.0 (4.0–10.0)
Distance to pectoralis muscle	4.0 (1.5–8.0)
Ablation power (W)	
20.0	4 (0.6%)
30.0	701 (96.7%)
40.0	18 (2.5%)
50.0	2 (0.3%)
Ablation energy (J)	1590.0 (900.0–2970.0)
Unit energy	10.14 (5.70–18.43)
Complications	
No	718 (99.0%)
Pricking	1 (0.1%)
Skin scalding	1 (0.1%)
Local erythema and swelling	2 (0.3%)
Fat liquefaction	3 (0.4%)

^a^Qualitative variables are summarized as frequency (percent) and quantitative variables are summarized as median (25th percentile – 75th percentile)

^b^Pathology was only recorded for 397 lesions while the other variables were recorded for all the 725 lesions

### MWA and outcomes

**Fig. 3:**
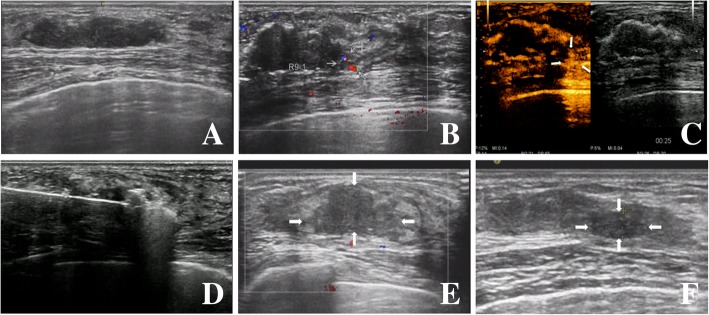
The conventional ultrasound before ablation (**a**), both the Color Doppler US and CEUS performed immediately after the first ablation indicated incomplete ablation (**b**, **c**), supplementary ablation (**d**), the lesion continued to shrink at the 3-month (**e**) and 12-month (**f**) follow up

MWA was performed at 30.0 W power in the majority of the lesions (96.7%) with the median energy used during the procedure being 1590 J and a median duration of 53 s. Before the MWA, the breast lesions presented as hypoechoic masses with well-defined boundaries. During the ablation, a hyperechoic region appeared around the antenna, visualized by intraprocedural grey-scale ultrasound. Immediately following ablation, lesions appeared as non-homogeneous hyperechoic masses with ill-defined boundaries. The 3 diameters of lesions were measured by combining grey-scale ultrasound images with those of CEUS. All lesions showed enhancement on CEUS/MRI before the microwave treatment. Half an hour after the MWA, 679/694 (97.8%) lesions showed no enhancement on CEUS/MRI, but 15 lesions with their largest diameter measuring greater than 2.5 cm presented with peripheral enhancement and subsequently underwent further supplementary ablation (Fig. 3a-f). Accordingly, complete ablation success rate for the first round of MWAs was 97.8%. Following the supplementary ablations, the success rate reached 100%, according to the CEUS or/and MRI. US- guided CNB was randomly performed on a few of the ablated lesions after the treatment and the histopathology findings showed various degrees of coagulation within these lesions. Most of the cases did not experience any complications after the procedure (99.0%) but ablation of one lesion was associated with pricking, another one with skin scalding, two lesions with local erythema and swelling, and 3 with fat liquefaction.

**Fig. 4:**
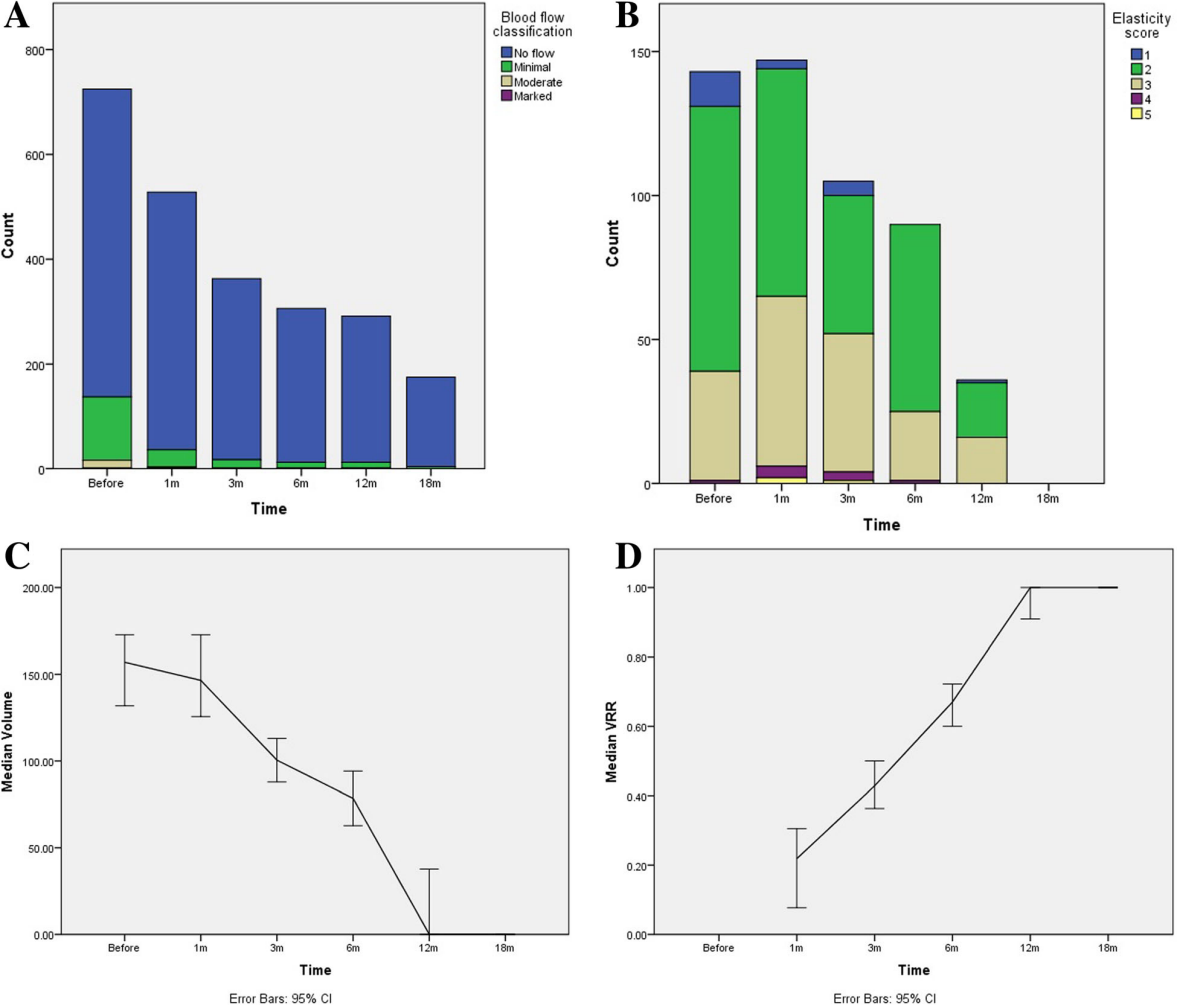
Trend of changes in (**a**) blood flow classification, (**b**) elasticity score, (**c**) lesions’ volume and (**d**) VRR after treatment

Table 2 and Fig. 4 show the changes in the blood flow classification, lesion’s volume, VRR, disappearance rate and elasticity score. Based on these results, the changes in all the variables were found to be statistically significant across different follow up time points. Blood flow classification and lesion’s volume showed a significant decrease after treatment, while both VRR and rate of disappearance significantly increased following treatment. At 18 months follow up, 93.0% of lesions were found to have disappeared. The elasticity score of the lesions did not follow a certain trend of changes and showed fluctuations along the different follow up intervals.

**Table 2:** Changes in blood flow classification, volume, VRR and elasticity score of the lesions after treatment

Variable^*^	Before treatment	1 month	3 months	6 months	12 months	18 months	*P* value^***^
Blood flow classification^**^	(*n* = 725)	(*n* = 528)	(*n* = 363)	(*n* = 306)	(*n* = 291)	(*n* = 175)	
No flow	588 (81.1%)	492 (93.2%)	346 (95.3%)	294 (96.1%)	279 (95.9%)	171 (97.7%)	< 0.001
Minimal	121 (16.7%)	33 (6.3%)	16 (4.4%)	11 (3.6%)	11 (3.8%)	4 (2.3%)	
Moderate	15 (2.1%)	2 (0.4%)	1 (0.3%)	1 (0.3%)	1 (0.3%)	0 (0.0%)	
Marked	1 (0.1%)	1 (0.2%)	0 (0.0%)	0 (0.0%	0 (0.0%)	0 (0.0%)	
	(*n* = 725)	(*n* = 529)	(*n* = 367)	(*n* = 311)	(n = 291)	(*n* = 185)	
Volume	157.0 (62.8–376.8)	146.5 (47.1–329.7)	100.5 (31.4–301.4)	78.5 (0.0–235.5)	0.0 (0.0–150.7)	0.0 (0.0–0.0)	< 0.001
VRR	–	0.2 (− 0.6–0.6)	0.4 (−0.2–0.8)	0.7 (0.1–1.0)	1.0 (0.5–1.0)	1.0 (1.0–1.0)	< 0.001
Disappearance rate	–	36 (6.8%)	47 (12.8%)	82 (26.4%)	151 (51.9%)	172 (93.0%)	< 0.001
Elasticity score	(*n* = 143)	(*n* = 147)	(*n* = 105)	(*n* = 90)	(n = 36)	(*n* = 0)	
1	12 (8.4%)	3 (2.0%)	5 (4.8%)	0 (0.0%)	1 (2.8%)	–	0.003
2	92 (64.3%)	79 (53.7%)	48 (45.7%)	65 (72.2%)	19 (52.8%)	–	
3	38 (26.6%)	59 (40.1%)	48 (45.7%)	24 (26.7%)	16 (44.4%)	–	
4	1 (0.7%)	4 (2.7%)	3 (2.9%)	1 (1.1%)	0 (0.0%)	–	
5	0 (0.0%)	2 (1.4%)	1 (1.0%)	0 (0.0%)	0 (0.0%)	–	

VRR: Volume Reduction Ratio

* Qualitative variables are summarized as frequency (percent) and quantitative variables are summarized as median (25th percentile – 75th percentile)

** Blood flow classification in this table is assessed by Color Doppler Flow Imaging (CDFI)

*** Chi-squared test and Kruskal Wallis test were used for qualitative and quantitative variables, respectively. *P* values represent the significance of differences in the assessed variables between different follow ups without taking the temporal relations into account

## Discussion

The considerable improvements in people’s living standards towards a higher quality of life have affected every aspect of their lives. In this regard, the treatment of breast lesions should not only aim to ensure survival of the patients, but rather should aim to preserve their function, address cosmetic concerns and be cognizant of the patients’ mental status throughout the whole process, particularly for benign tumors [4]. Accordingly, since traditional surgical treatments are associated with greater trauma to the tissues that could lead to formation of scars and eventually undesirable cosmetic outcomes, attention has been drawn to minimally invasive methods for treatment of benign breast tumors such as US-guided vacuum-assisted excision (US-VAE) and US-guided ablation [1].

Breast is the perfect organ for ablative treatments because it is superficial and only covered by the skin with no intervening structures, can be compressed in different directions to achieve the appropriate alignment of the instruments needed for ablation, and it can be monitored effectively by US [18]. Microwave ablation is currently being investigated in clinical practice as a potential treatment modality for benign breast lesions. This method uses thermal coagulation or protein denaturation to destroy the tissues in situ [19, 20]. Several studies have reported promising results for application of MWA in treatment of small breast lesions [21–25].

Congruent with current literature, the present study also illustrated the efficiency and safety of MWA in the treatment of benign breast lesions. Our results indicated that US-guided percutaneous MWA provides a complete ablation rate of 100% and a disappearance rate of up to 93.0% at 18 months after treatment, which was higher than that reported by Yu et al. and Zhou et al. [23, 25] and other thermal ablation techniques [26–30]. Our results showed that after treatment with MWA the lesions’ volume and blood flow significantly decreased in all cases and none of the patients experienced any considerable complications after treatment or had recurrence during the follow-ups.

The proposed mechanism for this treatment is that the high temperature generates a series of biochemical changes, such as tumor cell dehydration, intracellular protein denaturation and coagulation that brings the synthesis of tumor cells’ deoxyribonucleic acid and protein to a halt. It also destroys the blood supply of the lesion, which results in necrosis of tumor cells [31, 32].

Previous studies have reported the complete thermal ablation rate of breast lesions to be 61 to 97% [7, 33–35] . Similarly, in the present study complete ablation was not achieved in a few large or irregular lesions after the first round of treatment, and the rate for the first round was found to be within the range reported by previous studies. However, using the findings of post-ablation CEUS, supplementary treatment was performed for those lesions and the final complete ablation rate reached 100%. The incomplete ablation rate in our study could be attributed to the poor contrast of the images obtained from intraprocedural gray-scale ultrasound, which makes it difficult to accurately identify the spatial structure of the lesion and can lead to inaccurate positioning and navigation during the procedure. Particularly real-time monitoring of the tissues behind the hyperechoic areas can be difficult due to the changes caused by the procedure. In this regard, the echogenic changes on gray-scale ultrasound should only be used for rough approximation of the target area for ablation [36]. Hence, for a precise assessment of the complete ablation rate, contrast-enhanced imaging methods such as CE-MRI and CE-CT scan should be used within one week post-ablation [37, 38]. However, their high costs, inconvenience for immediate use after ablation and multiple acquisitions limit their application in assessment of ablation outcome in benign breast lesions. Some studies have reported that CEUS provides a similar diagnostic accuracy to that of CE-CT and CE-MRI [25, 39], and using CEUS assessment immediately after ablation can effectively reduce the residual of lesions in time and significantly improve the complete ablation rate [14, 40]. Therefore, in the present study all the patients underwent CEUS assessment before and after procedure, and only patients with large, multiple, irregular lesions and subjects with poor quality CEUS images underwent CE-MRI. To further improve the treatment outcome, we used the MST technique, which has been specifically recommended as the optimal option for the larger, irregular lesions and those located close to critical structures [16].

Another explanation for incomplete ablation of the lesions could be that in larger lesions the fiber usually lies within the lesion, which can limit the diffusion of heat leading to an uneven distribution of energy during the treatment. Also, the larger the lesion is, the more likely it is to be adjacent to other structures such as skin, nipple, chest wall or pectoral fascia, so in order to avoid injury of these normal tissues, the ablation range is set at a lower level, which decreases the efficacy of the treatment. One way to overcome this issue is injecting a solution around the lesion as a spacer to prevent injury to the surrounding tissues.

Previous studies have been mainly focusing on the complete ablation rate and post-operative complications and few reports are available on the long term follow up of these patients. In our series, we followed a large number of patients for a maximum of 18 months and found that the disappearance rate of lesions significantly increased over time. We also found a greater volume reduction in the lesions compared to some of the previously published reports [22, 41]. As was expected, larger lesions had a lower chance of disappearance after treatment.

Thermal ablation used in the RFA and MWA, leads to coagulative necrosis of the tissues mainly through heating water molecules. To our knowledge, normal breast tissue contains more fat and less water compared to breast tumors [42]. Therefore, MWA preferentially heats and damages high-water-content breast tumors, rather than the fatty and glandular components of the normal breast tissues [43]. Therefore, thermal ablation therapy usually has a low rate of complications, as observed in the present study. In order to yield complete ablation of a lesion while trying to avoid any complications, As Low As Reasonably Achievable (ALARA) principle should be used. The ALARA principle has been described in many clinical procedures such as radionuclide and contrast-enhanced exams, minimally invasive treatments, cosmetic surgeries, and so forth, to reduce the radiation exposure, side effects or any tissue injuries. The ALARA principle for minimally invasive treatment procedures such as ablation therapy, should be implemented through designing a detailed treatment plan in terms of time, power, shielding, etc. A timely procedure ensures that the intervention is effective while preventing any major traumas to the patient. Similarly, an appropriate power reduces treatment time and provides a higher chance of complete necrosis in the tumor tissues. Shielding protects against any injuries to the normal tissue and improves the cosmetic outcome of the procedure.

Currently, US-VAE is widely accepted as the superior technique for treatment of benign breast lesions. The complete resection rate for this method has been reported to range from 95 to 100% which is comparable to the success rate we found for the MWA technique in the present study [44–47]. Moreover, postoperative organized hematoma was not observed in any of the patients we treated with MWA, while this is one of the complications of the US-VAE. Therefore, it seems like the MWA could be considered as a good alternative for US-VAE and further studies are required to compare the efficacy of these two therapeutic methods with each other.

One of the limitations of the present study was the significant number of patients with limited follow up duration due to a recent MWA treatment, which limited our ability to perform robust analyses on the data acquired from these subjects. Moreover, the effects of different operators in the outcome were not taken into account, which could have provided valuable information given the operator-dependence of this treatment method. In this regard, further investigations with greater sample populations and longer follow ups are required to assess the value of MWA in the treatment of benign breast lesions and compare its results to other minimally-invasive procedures.

## Conclusions

Although surgery is currently considered as the common standard for the treatment of breast tumors, major attention has been drawn to minimally-invasive procedures owing to their cosmetically-appealing outcomes and cost-effectiveness. Among these methods, MWA treatment is shown to be safe and efficient, has the potential to be considered as a feasible alternative first line treatment option in the future. In this method microwaves are used to cause coagulative necrosis of the lesion which leads to shrinkage of the mass, or even its disappearance. It can also help with resolution of the patients’ symptoms.

## Abbreviations

ALARA: As Low As Reasonably Achievable
BI-RADS: Breast imaging reporting and data system
CE-MRI: Contrast-Enhanced Magnetic Resonance Imaging
CEUS: Contrast-enhanced ultrasound
CNB: Core needle biopsy
MST: Moving shot technique
MWA: Microwave ablation
RFA: Radiofrequency ablation
ROI: The region of interest
US: Ultrasound
US-VAE: Ultrasound-guided vacuum-assisted excision
VRR: The volume-reduction ratio
